# Supplementary figures and images for: Similarity between mutation spectra in hypermutated genomes of rubella virus and in SARS-CoV-2 genomes accumulated during the COVID-19 pandemic
Source: PLoS One. 2020 Oct 2;15(10):e0237689. doi: 10.1371/journal.pone.0237689 (PMC7531822; doi:10.1371/journal.pone.0237689)

**A.****ADAR1****S1 Figure**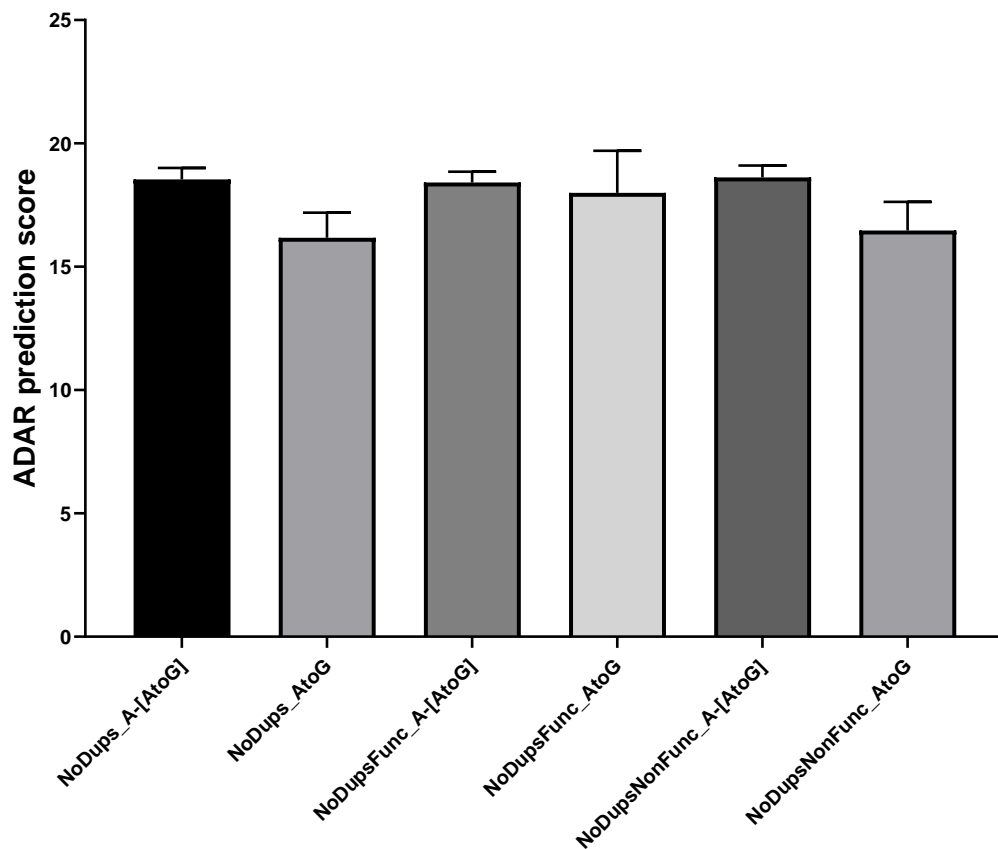**B.****ADAR2**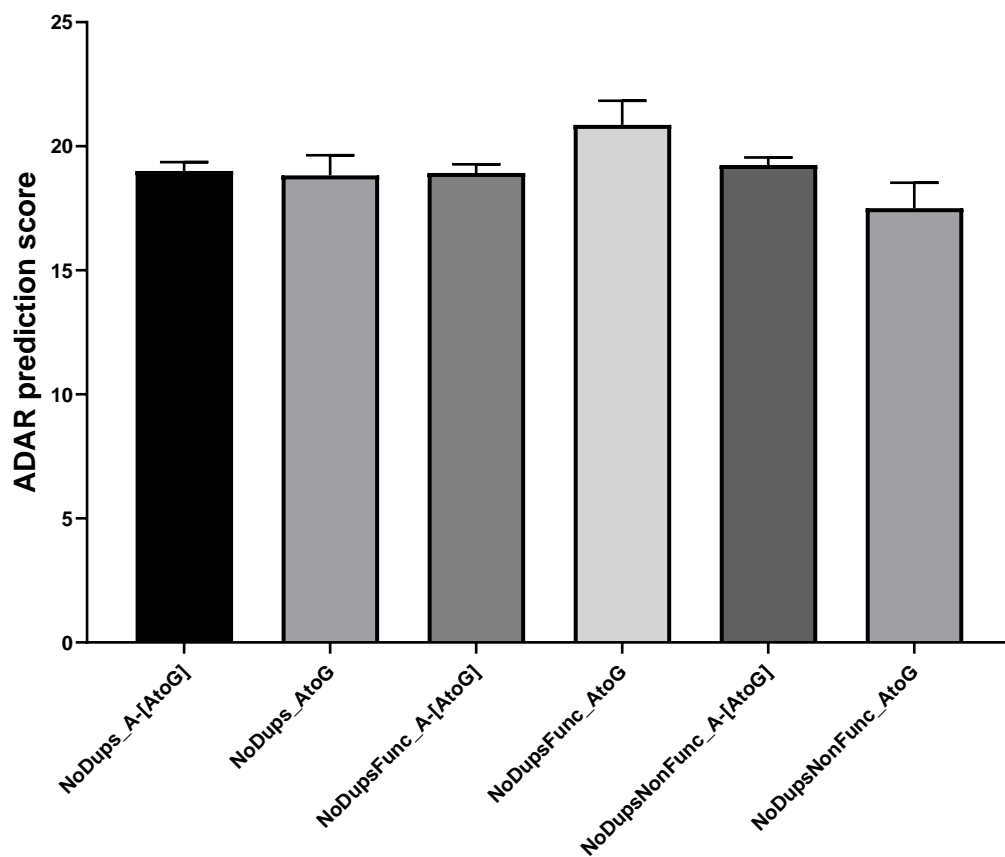

Supplement: S1 Fig — ADAR scores were calculated using the Web tool http://hci-bio-app.hci.utah.edu:8081/Bass/InosinePredict. Source data are in S1 Data. (PDF) [file pone.0237689.s001.pdf]

A.

NoDupsNonFunc\_tri\_C\_to\_U

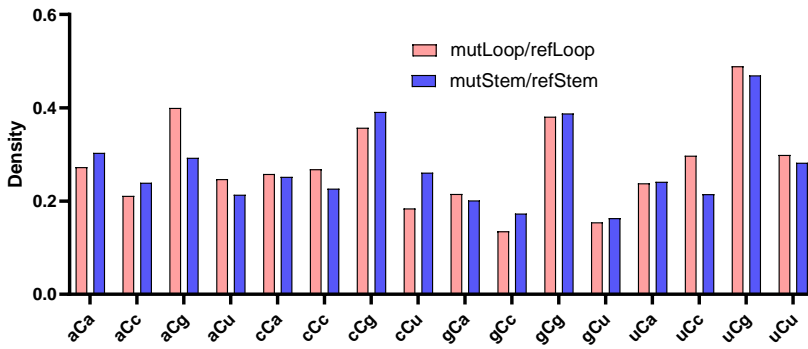

B.

NoDupsFunc\_tri\_C\_to\_U

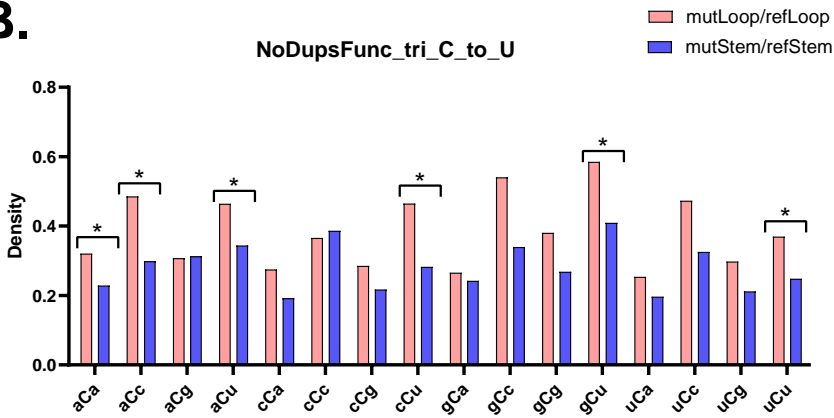

C.

NoDups\_tri\_C\_to\_U

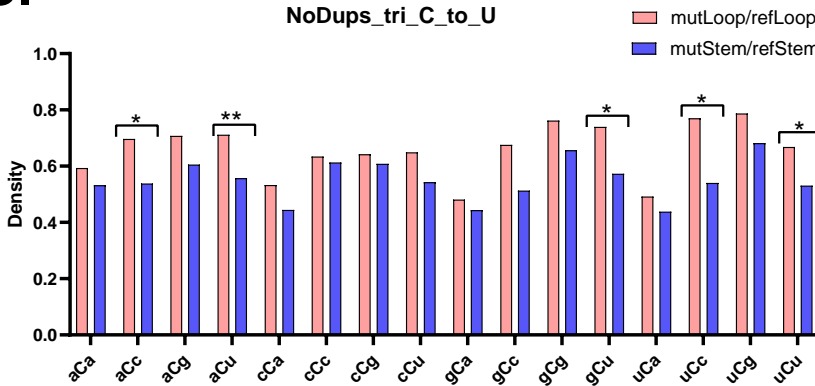

D.

Rubella\_tri\_C\_to\_U

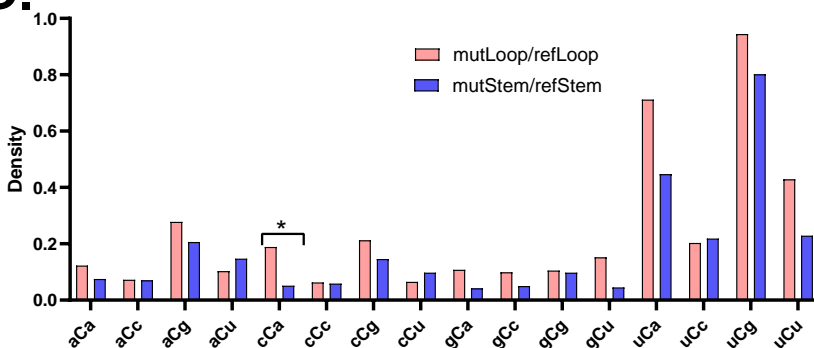

Supplement: S2 Fig — Bars represent densities of base substitutions in stem- or in loop-forming regions. Densities are calculated by dividing counts of each motif mutations in either loop or in stem by counts of this motif in the loop-forming or in stem-forming regions of the reference sequence. Statistical comparison between mutagenesis in stem vs loop for every base substitution was done by two-tailed Fisher’s exact test. P-values were corrected by FDR including 16 motifs containing C to U base substitution. Brackets indicate pairs passing FDR = 0.05. * <0.05, ** <0.005. Source data are in S7 Table. (PDF) [file pone.0237689.s002.pdf]

# A. NoDupsNonFunc\_tri\_G\_to\_U

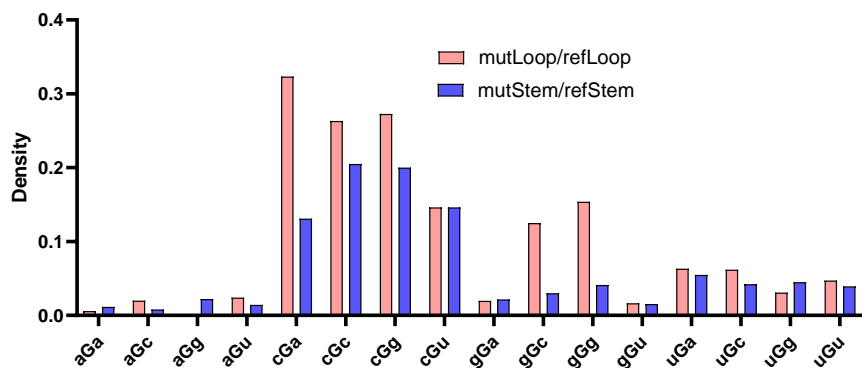

# B. NoDupsFunc\_tri\_G\_to\_U

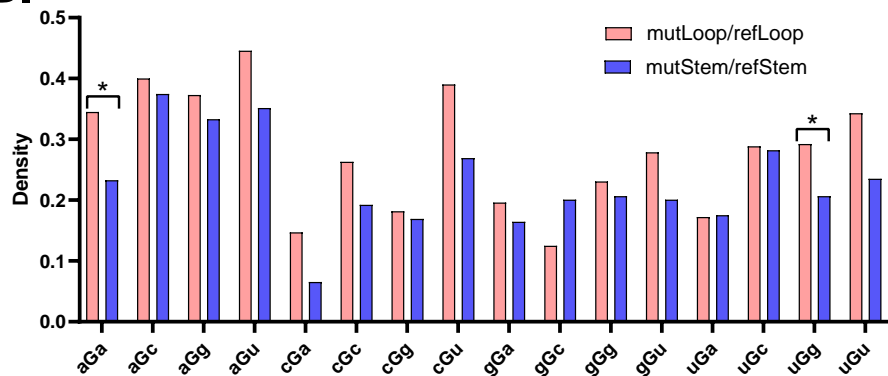

# C. NoDups\_tri\_G\_to\_U

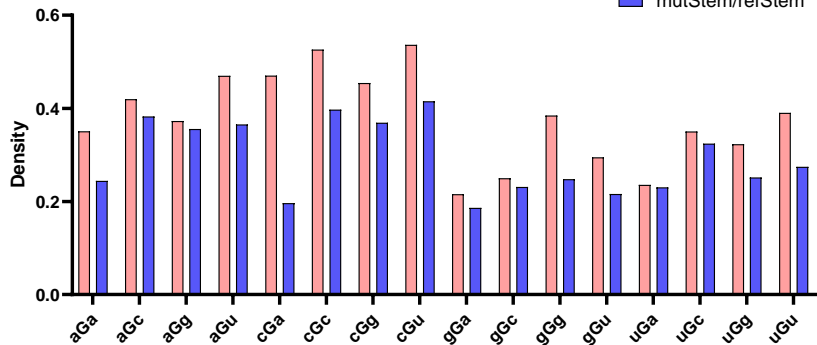

# D. Rubella\_tri\_G\_to\_U

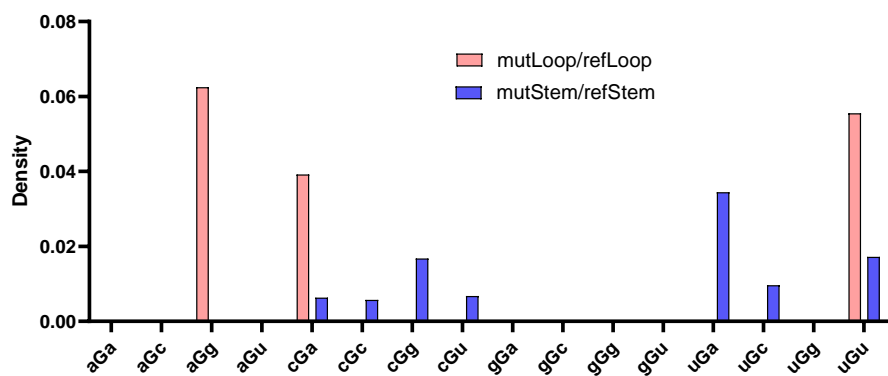

## S3 Figure

Supplement: S3 Fig — Bars represent densities of base substitutions in stem- or in loop-forming regions. Densities are calculated by dividing counts of each motif mutations in either loop or in stem by counts of this motif in the loop-forming or in stem-forming regions of the reference sequence. Statistical comparison between mutagenesis in stem vs loop for every base substitution was done by two-tailed Fisher’s exact test. P-values were corrected by FDR including 16 motifs containing G to U base substitution. Brackets indicate pairs passing FDR = 0.05. * <0.05. Source data are in S8 Table. (PDF) [file pone.0237689.s003.pdf]
