# Supplementary material for: Similarity between mutation spectra in hypermutated genomes of rubella virus and in SARS-CoV-2 genomes accumulated during the COVID-19 pandemic
Source: PLoS One. 2020 Oct 2;15(10):e0237689. doi: 10.1371/journal.pone.0237689 (PMC7531822; doi:10.1371/journal.pone.0237689)
Supplement: S1 Data — (ZIP) [file pone.0237689.s004.zip › S1_Data/Readme_S1_Data.docx]

**S1_Data. ADAR scores and ADAR analysis outputs for SARS-CoV-2 filtered MAFs**

**ADAR1_scores.xlsx – source data for S1 Figure**

Sets of ADAR1 score values for mutations in SARS-CoV-2 filtered MAFs; columns annotated in the Legend tab

**ADAR2_scores.xlsx – source data for S1 Figure**

Sets of ADAR2 score values for mutations in SARS-CoV-2 filtered MAFs; columns annotated in the Legend tab

**ADAR1_sites.txt
and
ADAR2_sites.txt**

Each row in the file corresponds a position in the complete reference sequence of SARS-CoV-2

| Column_name in files “*_sites.txt” | Column_content |
| --- | --- |
| V1 | Nucleotide in the plus-strand of the reference |
| V2 | ADAR score for a position with A in plus-strand;  “-1” - A in plus-strand separated by <5 nt from the end of reference;  “0” – other than A nucleotide in plus-strand of the reference |
| V3 | Nucleotide in the minus-strand of the reference |
| V4 | ADAR score for a position with A in minus-strand;  “-1” - A in minus-strand separated by <5 nt from the end of reference;  “0” – other than A nucleotide in minus-strand of the reference |
| Position | Position in the plus-strand of the reference |

***_anz4**

Each file contains ADAR scores for each nucleotide of the reference combined with information about mutations in each filtered MAF. Mutations in filtered MAFS are listed as in DNA (T standing for U) to allow direct comparison with DNA analysis outputs.

*

Columns in *_anz4_files

| Column_name | Column_content |
| --- | --- |
| Position | Position in reference |
| Start_position | Position of the first mutated nucleotide |
| End_position | Position of the last mutated nucleotide (same as Start for base substitutions_ |
| Reference_Allele | Nucleotide in reference |
| Tumor_Seq_Allele2 | Nucleotide introduced by mutation (column name retained from TCGA MAF format to allow smooth comparison of outputs) |
| V1 | Nucleotide in the plus-strand of the reference |
| V2 | ADAR score for a position with A in plus-strand |
| V3 | Nucleotide in the minus-strand of the reference |
| V4 | Position in the plus-strand of the reference |
